# Effects of the Great East Japan Earthquake on Secondary Sex Ratio and Perinatal Outcomes

**DOI:** 10.2188/jea.JE20150055

**Published:** 2016-02-05

**Authors:** Kohta Suzuki, Zentaro Yamagata, Miyuki Kawado, Shuji Hashimoto

**Affiliations:** 1 Department of Health Sciences, Interdisciplinary Graduate School of Medicine and Engineering, University of Yamanashi, Chuo, Yamanashi, Japan; 1 山梨大学大学院総合研究部医学域 社会医学講座; 2 Department of Hygiene, Fujita Health University School of Medicine, Toyoake, Aichi, Japan; 2 藤田保健衛生大学医学部 衛生学講座

**Keywords:** maternal stress, natural disaster, secondary sex ratio, gestational duration, birth weight, 母体ストレス, 自然災害, 出生性比, 妊娠期間, 出生体重

## Abstract

**Background:**

The effect of natural disasters on secondary sex ratio (SSR) and perinatal outcomes has been suggested. This study aimed to examine effects of the Great East Japan Earthquake on perinatal outcomes using vital statistics of Japan.

**Methods:**

Birth registration data from vital statistics of Japan between March 2010 and March 2012 were used. Pregnant women who experienced the earthquake were categorized according to their gestational period as of March 11, 2011, as follows: gestational weeks 4–11, 12–19, 20–27, and 28–36 (2011 group). Similarly, pregnant women who did not experience the earthquake were categorized according to their gestational period as of March 11, 2010 and used as controls (2010 group). We also categorized prefectures as “extremely affected”, “moderately affected”, and “slightly or unaffected” regions. SSR, birth weight, and gestational period were compared between both groups.

**Results:**

The number of singleton births was 688 479 in the 2010 group and 679 131 in the 2011 group. In the extremely affected region, the SSR among women at 4–11 weeks of gestation was significantly lower in the 2011 group compared with the 2010 group (49.8% vs 52.1%, *P* = 0.009). In the extremely affected region, children born to women who experienced the earthquake at 28–36 weeks of gestation had significantly lower birth weights.

**Conclusions:**

The SSR declined among women who experienced the earthquake during early pregnancy, particularly in the extremely affected region. However, no apparent negative effect of the earthquake on perinatal outcomes was observed, although birth weight of infants who were born to women who experienced the earthquake at 28–36 weeks of gestation were lower.

## Abstract (ja)

**背景：:**

自然災害が出生性比と周産期予後に影響を与えることが示唆されてきた。本研究では、東日本大震災が周産期予後に与える影響について、人口動態統計を用いて検討することを目的とした。

**方法：:**

2010年3月から2011年3月までの、人口動態統計出生票データを用いた。東日本大震災を経験した妊婦を、2011年3月11日時点での妊娠週数により妊娠4-11週、12-19週、20-27週、28-36週と分類した（2011年群）。2010年3月11日に妊娠していた女性を同様に分類し、コントロールとして用いた（2010年群）。さらに、都道府県を「深刻な影響を受けた地域」「中程度の影響を受けた地域」「わずかな影響、あるいはほとんど影響はなかった地域」という3群に分類した。出生性比、出生体重、妊娠期間を両群で比較した。

**結果：:**

2010年群では688,479人、2011年群では679,131人の単胎児が出生した。深刻な影響を受けた地域では妊娠4-11週に分類された妊婦で出生性比が2011年群で2010年群に比べて有意に減少していた（49.8% vs. 52.1%, *P* = 0.009）。また、深刻な影響を受けた地域では、妊娠28-36週で被災した妊婦は有意に出生体重が小さい児を出産していた。

**結論：:**

妊娠初期に被災した女性では、特に被害が深刻だった地域で、出生性比が減少していた。しかしながら、震災による周産期予後について、負の影響は明らかにならなかった。

## INTRODUCTION

The effect of natural disasters, such as earthquakes, on perinatal outcomes has been previously examined. For instance, in Chile, expectant mothers who experienced the strong earthquake in 2005 were found to be likely to deliver babies before full term, especially for female children. As a result, the ratio of males to females at birth (secondary sex ratio [SSR]) was decreased among these women.^1^ Further, in another report that examined the effects of the earthquake in Chile in 2010, Oyarzo et al reported that pregnant women who experienced the earthquake during early pregnancy were more likely to deliver low-birth-weight (LBW) infants than pregnant women who experienced the earthquake during late pregnancy.^2^ In contrast, a systematic review by Harville et al that examined the associations between natural disasters and perinatal outcomes suggested that women who experienced these disasters were likely to deliver small-for-gestational-age (SGA) infants,^3^ with a relatively small effect on gestational age. In Japan, Fukuda et al examined SSR after the Kobe earthquake in 1995; they concluded that at 9 months after the earthquake, the SSR was significantly lower than prior to the earthquake.^4^

It appears to be widely accepted that the SSR decreases following natural disasters. However, this effect is not fully understood. In addition, these previous studies had certain limitations. For example, although an effect of seasonality has been noted for SSR^5^^–^^7^ or preterm births,^8^ none of the above studies strictly adjusted for seasonal changes in perinatal outcomes. Further, studies using individual data for analysis had relatively small sample sizes, while studies with larger numbers of subjects were designed as ecological studies. Thus, it is difficult to determine the causal association between natural disasters and perinatal outcomes.

On March 11, 2011, a huge earthquake occurred in eastern Japan, called the Great East Japan Earthquake, inducing a subsequent massive tsunami. Millions of people were affected by this earthquake and tsunami, which was determined to be the most severe natural disaster in Japan in recorded history.^9^ By March 11, 2014, casualties included 15 884 deaths, 2633 missing, and 6148 injured, as confirmed by the National Police Agency of Japan.^10^ The most severely affected prefectures were Iwate, Miyagi, and Fukushima. Catalano et al examined the effect of this earthquake on SSR using birth registration data from the vital statistics data of Japan and suggested that sensitive periods during both early and late pregnancy led to a decreased SSR.^11^ Further, in the most severely affected prefectures, the rate of conception of males was reduced.^11^ However, these results were based on aggregate data, and these effects have not yet been examined on an individual level.

This study aimed to examine the effects of the Great East Japan Earthquake on SSR, birth weight, and gestational duration in weeks using individual birth registration data in the most severely affected prefectures and other prefectures in Japan.

## METHODS

### Birth registration data

In Japan, every birth must be registered by law. Thus, the study participants consisted of all births that occurred during the study period. The birth registration data are filed at relevant city health departments and entered into computerized files at the Ministry of Health, Labour and Welfare of Japan. Individual birth registration data from these vital statistics of Japan between March 2010 and March 2012 were used for this study. These data were anonymously provided under the Statistics Act in Japan and contain the birthplace, birth date, child’s sex, birth weight, gestational age, parity, and ages of the father and mother. Except for the ages of the parents, the obstetrician who attended the birth provided this information on the birth certificate. In this study, it was not necessary for participants to provide informed consent because every birth in Japan is legally required to be registered, and birth registration data are available for researchers to use with permission from the Ministry of Health, Labour and Welfare under the Statistics Act in Japan.

To examine the effect of the earthquake on SSR and perinatal outcomes, we compared two groups of pregnant women. Pregnant women who experienced the earthquake at 4–36 weeks of gestation were categorized according to their gestational period as of March 11, 2011, as follows: gestational weeks 4–11, 12–19, 20–27, and 28–36 (2011 group, *n* = 679 131). Pregnant women who did not experience the earthquake were similarly categorized according to their gestational period as of March 11, 2010, and used as controls (2010 group, *n* = 688 479). The gestational duration at the time of the earthquake was calculated by using the birth date and gestational age from the birth registration data. Because an effect of the earthquake on preterm birth and a gender difference in this effect have been suggested,^1^ women who experienced the earthquake after 37 weeks of gestation were excluded to examine the effect of earthquake on SSR and preterm birth. In addition, stillbirths were also excluded because the purpose of this study was only to examine the effect of the earthquake on live births. We also categorized prefectures as “extremely affected” (Iwate, Miyagi, and Fukushima; more than 100 dead or missing), “moderately affected” (more than 10 injured and less than or equal to 100 dead or missing), and “slightly or unaffected” (Figure).

**Figure.**
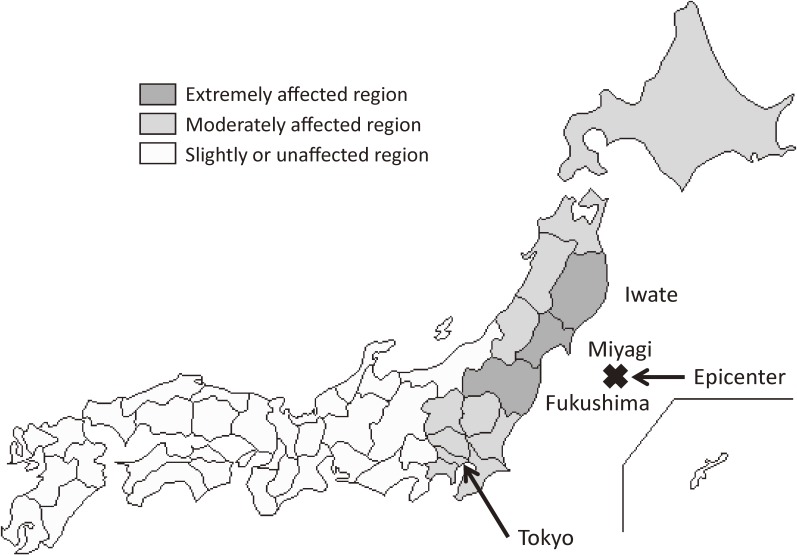
Categorization of prefectures by the extent of damage caused by the earthquake.

Because this study was conducted using anonymous data, which was provided under the Statistics Act in Japan, no ethical consideration was needed.

### Statistical analysis

To examine the effect of the earthquake on SSR and perinatal outcomes, we compared the above-mentioned 2011 and 2010 groups. Analyses were also conducted for the “extremely affected”, “moderately affected”, and “slightly or unaffected” regions. Further, only singleton babies were analysed because of known significant differences in gestational duration and birth weight between singleton and multiple births.^12^ First, the mean gestational duration, mean birth weight, mean maternal age at birth, mean birth order, prevalence of preterm birth, prevalence of LBW infants, and rate of first-born births were compared between groups in each region by sex. Student’s *t*-tests were used as crude analyses for mean gestational duration and mean birth weight. Chi-square tests were performed as crude analyses for categorical variables. Next, the SSRs of all births were compared between the 2010 and 2011 groups in each gestational period and region. Chi-square tests were conducted to analyse the differences. Finally, in each gestational period and region, multiple linear regression analyses were conducted to examine the effect of the earthquake on gestational duration after controlling for confounding factors, such as maternal age and parity. Similar analyses for birth weight were conducted to adjust for maternal age, parity, and gestational duration. In addition, based on the results of multiple regression analyses, adjusted mean gestational duration and birth weight were calculated by the least-squares method. These analyses were conducted in each gender because gender differences in effects of the earthquake have been suggested.^1^ All analyses were conducted using SAS version 9.3 (SAS Institute, Inc., Cary, NC, USA).

## RESULTS

### Study population

The characteristics of the study population are described in Table 1. The numbers of singleton babies born in 2010 and 2011 were 676 124 (348 040 boys and 328 084 girls) and 667 139 (342 344 boys and 324 795 girls), respectively. The mean maternal age at birth in the 2011 group was significantly higher than in the 2010 group in almost every region. Moreover, in the 2011 group, the mean birth order was significantly greater and the rate of first-born babies was lower than in the 2010 group. However, no significant differences in gestational duration, birth weight, prevalence of preterm birth, or rate of LBW infants were observed in the extremely affected region between the 2010 and 2011 groups.

**Table 1.** Population characteristics

	Extremely affected region						Moderately affected region						Slightly or unaffected region					
		
	Male		*P*^a^	Female		*P*^a^	Male		*P*^a^	Female		*P*^a^	Male		*P*^a^	Female		*P*^a^
					
	2010	2011		2010	2011		2010	2011		2010	2011		2010	2011		2010	2011	
Total	14 837	14 059		13 980	13 625		139 136	136 717		131 766	129 412		200 308	197 536		188 452	187 782	
(%)	51.5	50.8		48.5	49.2		51.4	51.4		48.6	48.6		51.5	51.3		48.5	48.7	
Singleton	14 583	13 817		13 763	13 389		136 735	134 515		129 389	127 162		196 722	194 012		184 932	184 244	
(%)	98.3	98.3		98.4	98.3		98.3	98.4		98.2	98.3		98.2	98.2		98.1	98.1	



Mean gestational duration (day)	274.9	274.8	0.6	275.8	275.6	0.14	274.2	274.2	0.9	275.4	275.3	0.047	274.5	274.4	0.006	275.6	275.6	0.9
SD	10.8	10.7		10.8	10.8		10.8	10.8		10.5	10.5		10.8	10.9		10.6	10.5	
(*n*)	14 583	13 817		13 763	13 389		136 735	134 515		129 389	127 162		196 722	194 012				

Preterm birth	705	625	0.2	524	513	0.9	6748	6664	0.8	4961	4961	0.7	9716	9814	0.09	7245	7003	0.07
(%)	4.8	4.5		3.8	3.8		4.9	5.0		3.8	3.8		4.9	5.1		3.9	3.8	
(*n*)	14 583	13 817		13 763	13 389		136 735	134 515		129 389	127 162		196 722	194 012		184 932	184 244	

Mean birth weight (g)	3071.9	3077.2	0.3	2984.4	2986.5	0.7	3062.9	3067.0	0.01	2978.4	2978.2	0.9	3063.9	3067.1	0.02	2976.7	2982.1	<0.0001
SD	414.3	410.3		401.5	403.2		414.1	414.2		397.7	397.1		416.6	419.3		401.3	401.0	
(*n*)	14 582	13 817		13 762.0	13 389		136 729	134 505		129 380	127 156		196 703	193 998		184 921	184 230	

Low-birth-weight infants	977	874	0.2	1201	1183	0.8	9532	9214	0.2	11 653	11 331	0.4	13 922	13 716	0.9	17 165	16 629	0.007
(%)	6.7	6.3		8.7	8.8		7.0	6.9		9.0	8.9		7.1	7.1		9.3	9.0	
(*n*)	14 582	13 817		13 762	13 389		136 729	134 505		129 380	127 156		196 703	193 998		184 921	184 230	

Mean maternal age at birth	29.9	30.1	0.02	29.9	30.2	<0.0001	31.1	31.2	<0.0001	31.1	31.2	<0.0001	30.4	30.5	<0.0001	30.4	30.5	<0.0001
SD	5.0	5.1		5.1	5.1		5.0	5.0		5.0	5.0		5.0	5.0		5.0	5.1	
(*n*)	14 583	13 817		13 763	13 389		136 735	134 515		129 389	127 162		196 722	194 012		184 932	184 244	

Mean birth order	1.78	1.80	0.07	1.77	1.81	0.0004	1.68	1.69	0.02	1.67	1.68	0.045	1.76	1.77	0.0002	1.76	1.78	<0.0001
SD	0.88	0.90		0.88	0.89		0.83	0.82		0.82	0.82		0.87	0.88		0.88	0.89	
(*n*)	14 583	13 817		13 763	13 389		136 735	134 515		129 389	127 162		196 722	194 012		184 932	184 244	

First-born	6516	6111	0.4	6299	5823	0.0002	68 410	66 566	0.005	65 077	63 220	0.003	90 918	88 579	0.0004	85 014	83 345	<0.0001
(%)	44.7	44.2		45.8	43.5		50.0	49.5		50.3	49.7		46.2	45.7		46.0	45.2	
(*n*)	14 583	13 817		13 763	13 389		136 735	134 515		129 389	127 162		196 722	194 012		184 932	184 244	

SD, standard deviation.

^a^*P* values were calculated by Chi-square test (categorical variables) and Student’s *t*-test (continuous variables).

### Analysis of the SSRs

In the extremely affected region, the male-to-female ratio among the women at 4–11 weeks of gestation was significantly lower in the 2011 group than in the 2010 group (49.8% in the 2011 group and 52.1% in the 2010 group; *P* = 0.009) (Table 2). However, the 2010 and 2011 groups did not significantly differ during other gestational periods. Further, in the moderately affected region, there was no significant difference between the 2010 and 2011 groups in each gestational period. In contrast, although the difference in SSR was relatively small, a significant decrease in SSR was noted between the 2010 and 2011 groups (51.7% vs 50.9%) among the women in the slightly or unaffected region at 4–11 weeks of gestation (*P* = 0.0011). However, there were no significant differences between the 2010 and 2011 groups during other gestational periods in this region.

**Table 2.** Comparison of secondary sex ratio (SSR) of all singleton babies between the 2010 and 2011 groups in each gestational period category and region

Gestational weeks	4–11 weeks			12–19 weeks			20–27 weeks			28–36 weeks		
			
	Total	Male	*P*^a^	Total	Male	*P*	Total	Male	*P*	Total	Male	*P*
Extremely affected region												
2010 group	6798	3541	0.009	7082	3680	0.66	6948	3499	0.07	7518	3863	0.07
SSR		52.1			52.0			50.4			51.4	
2011 group	6618	3298		6716	3465		6649	3450		7223	3604	
SSR		49.8			51.6			51.9			49.9	

Moderately affected region												
2010 group	66 757	34 155	0.97	65 525	33 949	0.86	65 018	33 251	0.28	68 824	35 380	0.61
SSR		51.2			51.8			51.1			51.4	
2011 group	65 137	33 320		64 947	33 617		63 721	32 780		67 872	34 798	
SSR		51.2			51.8			51.4			51.3	

Slightly or unaffected region												
2010 group	94 792	48 980	0.0011	94 700	48 466	0.43	92 986	48 127	0.21	99 176	51 149	0.48
SSR		51.7			51.2			51.8			51.6	
2011 group	93 363	47 541		94 709	48 641		91 825	47 257		98 359	50 573	
SSR		50.9			51.4			51.5			51.4	

^a^*P* values were calculated by Chi-square test.

### Linear regression models for gestational duration and birth weight

No significant decline in gestational duration was observed in the 2011 group compared with the 2010 group for either sex (Table 3). However, in the extremely affected region, a significant decrease in birth weight was observed for male children of women who had been at 28–36 weeks of gestation during the earthquake compared with the 2010 group. However, there was no significant decrease in birth weight in the 2011 group compared with the 2010 group among women in other gestational periods. Further, in the moderately affected and the slightly or unaffected region, no significant decline in the gestational duration or birth weight was noted in the 2011 group compared with the 2010 group for either sex.

**Table 3.** Adjusted gestational duration and birth weight for each gestational period category and region

Gestational weeks	4–11 weeks								12–19 weeks								20–27 weeks								28–36 weeks							
			
Male	Gestational duration (days)				Birth weight (g)				Gestational duration (days)				Birth weight (g)				Gestational duration (days)				Birth weight (g)				Gestational duration (days)				Birth weight (g)			

	Coeff.	SE	*P*	Adjusted means^a^	Coeff.	SE	*P*	Adjusted means^b^	Coeff.	SE	*P*	Adjusted means^a^	Coeff.	SE	*P*	Adjusted means^b^	Coeff.	SE	*P*	Adjusted means^a^	Coeff.	SE	*P*	Adjusted means^b^	Coeff.	SE	*P*	Adjusted means^a^	Coeff.	SE	*P*	Adjusted means^b^
Extremely affected region																																
2010 group				275.06				3075.19				274.81				3061.61				274.57				3065.34				275.10				3083.06
2011 group	−0.40	0.27	0.13	274.66	15.19	8.11	0.06	3090.38	−0.29	0.27	0.28	274.52	5.46	7.79	0.48	3067.07	0.43	0.26	0.10	275.00	21.61	8.08	0.008	3086.95	0.12	0.22	0.58	275.22	−15.78	7.68	0.04	3067.29
(*n*)	6839				6839				7145				7145				6949				6948				7467				7467			

Moderately affected region																																
2010 group				274.01				3057.32				274.04				3057.17				274.00				3058.20				274.84				3079.37
2011 group	0.16	0.09	0.06	274.18	6.45	2.55	0.011	3063.77	0.02	0.09	0.85	274.06	2.40	2.55	0.35	3059.58	0.01	0.08	0.89	274.02	5.53	2.60	0.03	3063.73	−0.09	0.07	0.21	274.75	−0.06	2.54	0.98	3079.31
(*n*)	67 475				67 469				67 566				67 561				66 031				66 027				70 178				70 177			

Slightly or unaffected region																																
2010 group				274.34				3057.77				274.29				3053.75				274.38				3063.30				275.00				3077.33
2011 group	−0.08	0.07	0.29	274.26	3.61	2.15	0.09	3061.38	−0.08	0.07	0.27	274.21	6.50	2.12	0.002	3060.26	−0.10	0.07	0.14	274.28	0.86	2.18	0.69	3064.16	−0.02	0.06	0.72	274.98	7.40	2.12	0.0005	3084.73
(*n*)	96 521				96 511				97 107				97 105				95 384				95 373				101 722				101 712			

Gestational weeks	4–11 weeks								12–19 weeks								20–27 weeks								28–36 weeks							
			
Female	Gestational duration (days)				Birth weight (g)				Gestational duration (days)				Birth weight (g)				Gestational duration (days)				Birth weight (g)				Gestational duration (days)				Birth weight (g)			

Extremely affected region																																
2010 group				275.67				2985.11				275.90				2977.79				275.32				2967.09				276.22				3002.96
2011 group	−0.12	0.28	0.68	275.55	−6.41	8.10	0.43	2978.70	−0.50	0.27	0.07	275.40	9.92	8.15	0.22	2987.71	0.09	0.27	0.75	275.41	19.26	7.90	0.015	2986.35	0.09	0.21	0.68	276.31	−7.08	7.71	0.36	2995.88
(*n*)	6577				6577				6653				6653				6648				6648				7274				7273			

Moderately affected region																																
2010 group				275.25				2973.89				275.22				2972.48				275.19				2975.67				275.72				2988.95
2011 group	−0.07	0.09	0.39	275.18	1.17	2.56	0.65	2975.06	0.04	0.09	0.64	275.26	0.10	2.57	0.97	2972.58	−0.04	0.08	0.61	275.15	−1.16	2.62	0.66	2974.51	−0.11	0.07	0.13	275.62	3.01	2.54	0.24	2991.96
(*n*)	64 419				64 417				62 906				62 899				62 708				62 703				66 518				66 517			

Slightly or unaffected region																																
2010 group				275.45				2971.83				275.36				2968.05				275.42				2975.67				276.04				2992.07
2011 group	−0.04	0.07	0.59	275.41	6.18	2.17	0.004	2978.01	0.03	0.07	0.68	275.39	3.07	2.15	0.15	2971.13	0.15	0.07	0.04	275.57	5.24	2.19	0.02	2980.90	−0.03	0.06	0.62	276.01	3.94	2.14	0.07	2996.01
(*n*)	91 634				91 629				92 302				92 291				89 427				89 422				95 813				95 809			

^a^Adjusted for maternal age and parity and calculated by least-squares method.

^b^Adjusted for maternal age, parity, and gestational duration and calculated by least-squares method.

## DISCUSSION

This study is the first to examine the effects of the Great East Japan Earthquake on SSR and perinatal outcomes, with consideration of the seasonal patterns of perinatal indicators. Our results suggested that SSR declined in women who experienced the earthquake during their early pregnancy, particularly for those in the extremely affected region. For perinatal outcomes, such as gestational duration and birth weight, no significant negative effects of the earthquake were observed in the moderately affected or slightly affected regions; however, in the extremely affected region, adjusted mean birth weight was smaller for male children delivered by women who experienced the earthquake at 28–36 weeks of gestation.

Some demographic differences were noted between the 2010 and 2011 groups. However, the total fertility rates of women during these years were similar.^13^ In addition, these differences were very small, although some were significant because of the huge sample size. In fact, there appeared to be few significant effects of the earthquake on perinatal outcomes. Thus, these differences, particularly the difference in SSR, might not be associated with the earthquake. Moreover, although SSR in the 2011 group also significantly decreased in the slightly or unaffected regions compared with the 2010 group, the effect of the earthquake on SSR may have been limited in the extremely affected region. The difference in SSR between the slightly and unaffected region (51.7% in the 2010 group vs 50.9% in the 2011 group among women at 4–11 weeks of gestation) was statistically significant; however, no such significant difference was observed in the extremely affected region. In the extremely affected region, there was no significant difference in SSR among women who experienced the earthquake at 20–27 and 28–36 weeks of gestation, although these differences were larger than the differences among the women at 4–11 weeks of gestation in the slightly or unaffected region. We believe that the statistically significant difference seen in the slightly or unaffected region could merely be a result of the large study population. Therefore, it is difficult to assess whether or not this difference was a result of the earthquake.

With respect to SSR, our results were largely consistent with those of previous studies. Catalano et al examined the effects of the same earthquake and concluded that rate of male births decreased because of decreased conception rates of male foetuses and increased selection of female foetuses *in utero*.^11^ However, they only examined the number of births in each month, since they analysed aggregate birth registration data. Thus, their results did not consider the stressors of the earthquake at the individual level. Despite differing methodologies, our results concerning the effects of the earthquake during early pregnancy were consistent with their findings. Further, our results, which suggested that experiencing the earthquake during early pregnancy was associated with decreased SSR, were largely consistent with the results of Fukuda et al, although they also analysed aggregated birth registration data concerning the Kobe earthquake.^4^ However, our findings regarding the effect of the earthquake during late pregnancy differed somewhat from those of Catalano et al.^11^ Where Catalano et al suggested that there might be a sensitive period in late gestation, our results suggested that SSR was not significantly different and that the birth weight in males was likely to be lower when the mothers experienced the earthquake at 28–36 weeks of gestation. However, in our analysis, it was impossible to assess the effect of the earthquake after 37 weeks of gestation because we excluded women who experienced the earthquake after that point from the analysis. Therefore, in women who experienced the earthquake after 37 weeks of gestation, it was suggested by the previous reports that the gestational duration for male children was likely to be shortened because of intrauterine growth restriction,^14^ with a resultant decline in SSR during late pregnancy. However, because the adjusted difference in mean birth weight between 2010 and 2011 was only 16 g, the effect of the earthquake on foetal growth might be small.

The present findings were also largely consistent with results of a study by Torche and Kleinhaus concerning the impact on SSR of the 2005 earthquake in Chile, which analysed individual birth registration data and concluded that the SSR decreased among women who experienced that earthquake.^1^ They also demonstrated that expectant mothers who experienced that earthquake were likely to deliver babies before full term, especially for female children,^1^ although we noted no significant effect of the earthquake on shortened gestational duration. However, only in Iwate Prefecture, which is part of the extremely affected region, a significant decrease in gestational duration for female children was observed among women who experienced the earthquake during early pregnancy (data not shown). Therefore, our results in this prefecture were similar to their results although the number of population in this prefecture was limited.

It has been suggested that cohorts that have experienced severe maternal stress during pregnancy, such as that caused by natural disasters, have lower-than-expected SSRs.^15^^,^^16^ Natural selection is hypothesized to include conservation mechanisms by which stressed pregnant females preferentially cull frail male foetuses. This hypothesis, known as the Trivers-Willard hypothesis,^17^ proposes that adverse maternal health shocks around conception or during pregnancy lead to a sex ratio skewed in favour of female offspring, and the present results support this hypothesis. One possible explanation for the hypothesis is that, because male foetuses grow faster and larger than female foetuses,^18^ male foetuses may require more resources from the mother and may be less likely to adapt their development to a stressful intrauterine environment.^1^ On the other hand, female foetuses might need fewer resources than males for development *in utero*^1^ and may show reduced growth and demands on the mother in response to maternal stress.^19^ Finally, male newborns are also more likely to die than females.^20^

Previous studies regarding the effects of earthquakes on perinatal outcomes, such as the report regarding the Chile earthquake, have indicated that expectant mothers who experience the earthquake during early pregnancy are likely to have preterm deliveries.^2^ In our analyses, some negative effects of the earthquake on gestational duration and birth weight were only observed in part of the extremely affected region. Moreover, no clear effect of the earthquake on perinatal outcomes was observed. This discrepancy between the results of our study and those of the study conducted in Chile might be caused by differences in geographical factors and medical support systems after natural disasters in each country.

This study has several limitations that warrant mention. First, to control for the seasonality of SSR and gestational duration, women who were pregnant at 1 year before the earthquake were used as controls. It is possible that this interval was not adequate to control for seasonality; however, very few studies have previously considered the seasonality of SSR and perinatal outcomes. Second, since Japanese birth registration data do not include information regarding maternal lifestyle and complications during pregnancy, it was difficult to adjust for potential confounders of perinatal outcomes, such as maternal smoking during pregnancy; however, we did control for maternal age at birth and birth order. In addition, the method of calculating gestational age was not specified in the vital statistics, although information concerning gestational age was provided by obstetricians. In any case, the method for determining gestational age is likely to have been the same in 2010 and 2011 as well as between each gestational week category of participants and among regions, because there is a guideline for obstetrical practice in Japan.^21^ In addition, this kind of measurement error is non-differential. As a result, this study limitation would be expected to reduce the statistical power of comparison between 2010 and 2011 groups. Thus, it is unlikely that detected differences in SSR were due to chance, as non-differential errors generally make variance of distribution larger. Particularly in the analyses of perinatal outcomes, there might be certain type I errors due to multiple comparisons because many comparisons were made between the 2010 and 2011 groups in each gestational period and region. However, for the comparison of SSR in the extremely affected region, we expect our results to be robust because the *P* value was very small. Further, since the earthquake, emigration from the disaster-stricken areas has increased, particularly from Fukushima Prefecture.^22^ In addition, the number of deliveries, including deliveries of women who went to their parents’ home and delivered at a hospital in their parents’ town to obtain perinatal support from their parents (which is called a “Satogaeri” delivery), decreased in the extremely affected region after the earthquake due to problems of gaining access to medical facilities because of the shutdown of some facilities.^23^ These factors may have resulted in differences between the 2010 and 2011 groups. However, the effect of population movement might be limited because there was no significant difference between the 2010 and 2011 groups in other areas, though some people migrated from the extremely affected region. Further, it was impossible to examine differences in the effects between the coastal areas, which were affected by the tsunami, and other areas. The amount of data we obtained from municipalities in the coastal and mountainous areas was relatively small. Therefore, we could not differentiate between the effects of the earthquake versus those of the tsunami. However, if only the effects of the tsunami existed, the results would be underestimated because the effects of the tsunami was very few in other areas. Finally, we did not examine the effect of the earthquake on rates of abortions and stillbirths, which might be essential to clarify the mechanism of differences in SSR. However, our results support previous hypotheses about the mechanism of such differences that do not rely on abortions and stillbirths, since the present findings were mostly consistent with those of previous studies.

Despite these limitations, we consider our results to be valuable for examining the biological mechanisms underlying the effects of natural disasters on SSR and perinatal outcomes, particularly since this study analysed huge datasets from national individual birth registration records.

In conclusion, this study is the first to analyse the effects of the Great East Japan Earthquake on perinatal outcomes using individual data, with consideration of the seasonal patterns of perinatal indicators. Our findings suggest that SSR declined among women who experienced the earthquake during early pregnancy, particularly in the extremely affected region. However, with regard to perinatal outcomes, such as gestational duration and birth weight, no apparent negative effect of the earthquake was observed, although birth weight of infants who were born to women who experienced the earthquake at 28–36 weeks of gestation were lower.

## ONLINE ONLY MATERIAL

Abstract in Japanese.
