# Supplementary material for: Effects of the Great East Japan Earthquake on Secondary Sex Ratio and Perinatal Outcomes
Source: J Epidemiol. 2016 Feb 5;26(2):76–83. doi: 10.2188/jea.JE20150055 (PMC4728118; doi:10.2188/jea.JE20150055)
Supplement: Abstract in Japanese. [file je-26-076-s001.pdf]

# 東日本大震災が出生性比、周産期予後に与えた影響

鈴木　孝太[1]、山縣然太朗[1]、川戸美由紀[2]、橋本　修二[2]
1　山梨大学大学院総合研究部医学域　社会医学講座
2　藤田保健衛生大学医学部　衛生学講座

**背景 :** 自然災害が出生性比と周産期予後に影響を与えることが示唆されてきた。本研究では、東日本大震災が周産期予後に与える影響について、人口動態統計を用いて検討することを目的とした。
**方法 :** 2010 年 3 月から 2011 年 3 月までの、人口動態統計出生票データを用いた。東日本大震災を経験した妊婦を、2011 年 3 月 11 日時点での妊娠週数により妊娠 4-11 週、12-19 週、20-27 週、28-36 週と分類した（2011 年群）。2010 年 3 月 11 日に妊娠していた女性を同様に分類し、コントロールとして用いた（2010 年群）。さらに、都道府県を「深刻な影響を受けた地域」「中程度の影響を受けた地域」「わずかな影響、あるいはほとんど影響はなかった地域」という 3 群に分類した。出生性比、出生体重、妊娠期間を両群で比較した。
**結果 :** 2010 年群では 688,479 人、2011 年群では 679,131 人の単胎児が出生した。深刻な影響を受けた地域では妊娠 4-11 週に分類された妊婦で出生性比が 2011 年群で 2010 年群に比べて有意に減少していた（49.8% vs. 52.1%, $P = 0.009$）。また、深刻な影響を受けた地域では、妊娠 28-36 週で被災した妊婦は有意に出生体重が小さい児を出産していた。
**結論 :** 妊娠初期に被災した女性では、特に被害が深刻だった地域で、出生性比が減少していた。しかしながら、震災による周産期予後について、負の影響は明らかにならなかった。

キーワード : 母体ストレス、自然災害、出生性比、妊娠期間、出生体重
